# The Extraction of Coupling-of-Modes Parameters in a Layered Piezoelectric Substrate and Its Application to a Double-Mode SAW Filter

**DOI:** 10.3390/mi14122205

**Published:** 2023-12-03

**Authors:** Lingqi Li, Qiaozhen Zhang, Yang Yang, Baichuan Li, Yahui Tian, Xiangyong Zhao, Sulei Fu

**Affiliations:** 1 The College of Information, Mechanical and Electrical Engineering, Shanghai Normal University, Shanghai 200233, China; 1000446645@smail.shnu.edu.cn (L.L.); yyang@shnu.edu.cn (Y.Y.); 1000494740@smail.shnu.edu.cn (B.L.); xyzhao@shnu.edu.cn (X.Z.); 2 State Key Laboratory of Acoustics, Institute of Acoustics, Chinese Academy of Sciences, Beijing 100190, China; 3 The Key Laboratory of Advanced Materials (MOE), School of Materials Science and Engineering, Tsinghua University, Beijing 100084, China; fusulei@mail.tsinghua.edu.cn

**Keywords:** surface acoustic wave, coupling of modes, layered structure, finite element method

## Abstract

This paper presents an advanced method that combines coupling-of-modes (COM) theory and the finite element method (FEM), which enables the quick extraction of COM parameters and the accurate prediction of the electroacoustic and temperature behavior of surface acoustic wave (SAW) devices. For validation, firstly, the proposed method is performed for a normal SAW resonator. Then, the validated method is applied to analysis of an I.H.P. SAW resonator based on a 29°YX−LT/SiO_2_/SiC structure. Via optimization, the electromechanical coupling coefficient (*K*^2^) is increased up to 13.92% and a high quality (*Q*) value of 1265 is obtained; meanwhile, the corresponding temperature coefficient of frequency (TCF) is −10.67 ppm/°C. Furthermore, a double-mode SAW (DMS) filter with low insertion loss and excellent temperature stability is also produced. It is demonstrated that the proposed method is effective even for SAW devices with complex structures, providing a useful tool for the design of SAW devices with improved performance.

## 1. Introduction

Surface acoustic wave (SAW) devices have been widely used in wireless communication systems; particularly, filters and duplexers based on SAW resonators are mass-produced and applied in the radio frequency (RF) front-end due to their advantages of high isolation, low insertion loss, etc. With the advent of the 5G era, there is a growing demand for SAW devices in terms of their high frequency, large electromechanical coupling coefficient (*K^2^*), high quality (*Q*), good temperature coefficient of frequency (TCF) etc.

The performance of SAW devices mainly depends on their piezoelectric substrates. A normal SAW devices on bulk piezoelectric single-crystal materials such as lithium tantalate (LiTaO_3_, LT) and lithium niobate (LiNbO_3_, LN) have a limitation to their high frequency and *Q* factor [1,2,3,4,5,6,7,8,9]. To solve these challenges, T. Takai et al. proposed the I.H.P. SAW device based on a LiTaO_3_/SiO_2_/AlN/Si multi-layered structure and demonstrated that the resonators employing I.H.P. SAW device offers higher Bode-Q values of over 6000 at 0.9 GHz to1900 at 3.5 GHz and a very small TCF of −8 ppm/°C [4]. Because of increasingly stringent requirements for performance enhancement, techniques for the analysis and design of SAW devices on such layered piezoelectric substrates with more complex structures are called for [10,11].

In the available numerical simulation techniques, the coupling-of-modes (COM) model is commonly used and proven to be an efficient modeling approach to design SAW devices [10,12,13,14,15]. However, the COM model is a phenomenological model, and its COM parameters have to be first determined either via measurements or precise theoretical numerical methods, for example the finite element method (FEM), or the boundary element method combined with a finite element method (FEM/BEM) [16,17,18,19,20,21]. Many SAW researchers have made efforts to precisely extract COM parameters. V. Plessky et al. proposed a two-parameter model that approximates the dispersion of leaky waves by characterizing the dispersion relationship of pure surface shear waves propagating in a Bragg grating within the forbidden band [22]. B. P. Abbott and K. Hashimoto et al. further combined the V. Plessky dispersion relation with the COM theory, establishing the STW-COM model and providing a method for extracting STW-COM parameters [23]. The current COM models take into account phenomena including the excitation, propagation, and scattering of surface acoustic waves, and simulate normal SAW devices well. Nevertheless, temperature-dependent COM parameter extraction is still absent. 

Therefore, this work proposes a new method combining the COM model with multiphysics quasi-3D periodic FEM model considering the temperature field, which enables the quick and accurate prediction of electroacoustic properties and temperature behavior for both normal and layered SAW devices. For validation, the admittances of two resonators based on 36°YX−LT and 36°YX−LT/SiO_2_/SiC are calculated by using the *P*-matrix with the extracted COM parameters. Then, the proposed method is employed in the simulation of a full-sized double-mode SAW (DMS) filter, and its temperature behavior is studied and discussed. Furthermore, a design of a high-performance DMS filter with low insertion loss and improved temperature stability is achieved.

## 2. Experimental Methods

### 2.1. COM Theory and P-Matrix Model

**Figure 1:**
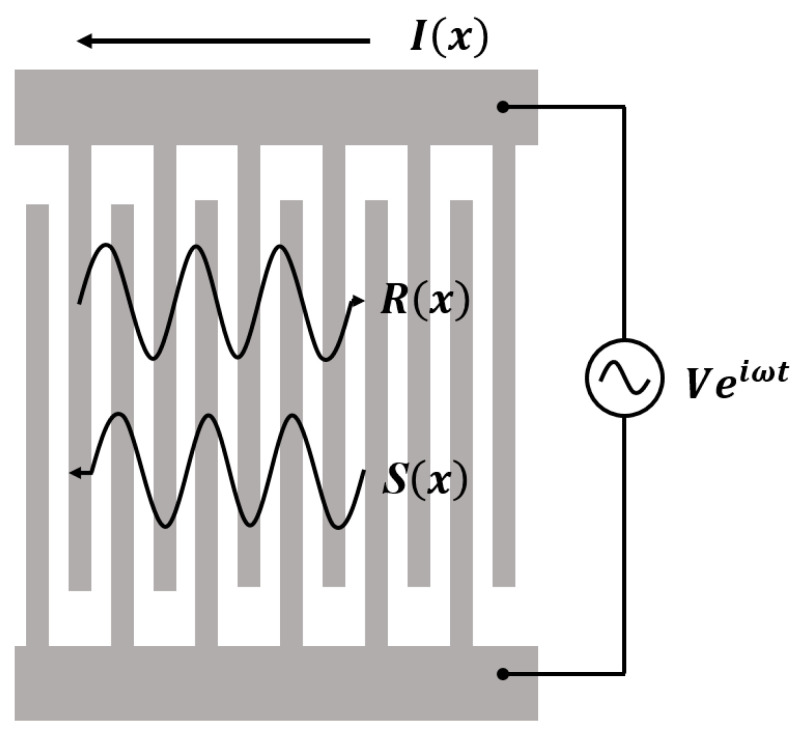
Propagation of SAW in periodic structure.

The materials in Figure 1 depict a basic SAW transducer structure. The COM model is widely employed to investigate the coupling behavior of two counter-propagating acoustic waves in a periodic grating structure. 

It is assumed that there exist two counter-propagating acoustic wave modes *R*(*x*) and *S*(*x*) in an infinitely long periodic grating array. These modes couple to each other through the interdigital reflection effect of the metal grating. Additionally, an external excitation source alternating the voltage, *V*, on the transducer stimulates acoustic waves, which perturbs the forementioned wave modes. The existence of electrodes on the surface affects the SAW velocity of the free surface and introduces mode coupling. Assuming linear coupling between amplitude, voltage, and current, the COM equations can be written as follow [10]:

(1) $$\left\{\begin{array}{c} \frac{dR\left(x\right)}{dx}=-i∆R\left(x\right)+i\kappa S\left(x\right)+i\alpha V,\\ \frac{dS\left(x\right)}{dx}=-i\kappa^{*}R\left(x\right)+i∆S\left(x\right)-i\alpha^{*}V,\\ \frac{dI\left(x\right)}{dx}=-2i\alpha^{*}R\left(x\right)-2i\alpha S\left(x\right)+i\omega CV, \end{array}\right.$$

where *κ* represents the coupling coefficient, α signifies the excitation coefficient, *C* indicates the static capacitance, and ∆ denotes the detuning parameter, which is defined by the following:

(2) $$∆=\frac{\omega }{v}-k_{0}-j\gamma ,\;$$

where ω is given by ω = 2π*f*, in which *f* is the frequency of the two waves. *v* denotes the effective SAW velocity, $k_{0}$ indicates propagation wave number, and *γ* represents attenuation. In Equation (1), the five independent variables including *v*, *κ*, *α*, *γ*, and *C* are COM parameters that need to be determined.

Under the linear assumption, the transducer can be equivalently represented by a three-port matrix, which consists of two acoustic ports and one electrical port. Therefore, the solutions to the equations of COM can be obtained using the *P*-matrix [10]:

(3) $$\left[\begin{array}{c} b_{1}\\ b_{2}\\ I \end{array}\right]=\left[\begin{array}{ccc} P_{11} & P_{12} & P_{13}\\ P_{21} & P_{22} & P_{23}\\ P_{31} & P_{32} & P_{33} \end{array}\right]\left[\begin{array}{c} a_{1}\\ a_{2}\\ V \end{array}\right].\;$$

The matrix only considers the physical quantities at its boundaries, including the incident acoustic waves (*a*_1_ and *a*_2_), the outgoing acoustic waves (*b*_1_ and *b*_2_), the input voltage *V*, and the current *I* flowing into it. Assuming the COM parameters are known, the *P*-matrix unit can be calculated using the following equation [10]:

(4) $$\begin{array}{l} P_{11}=\frac{j\kappa^{*}sin\left(LD\right)}{Dcos\left(LD\right)\;\;+\;\;j\Delta sin\left(LD\right)}\\ P_{12}=P_{21}=\frac{{\left(-1\right)}^{2N}D}{Dcos\left(LD\right)\;+\;j\Delta sin\left(LD\right)}\\ P_{22}=\frac{j\kappa sin\left(LD\right)}{Dcos\left(LD\right)\;+\;j\Delta sin\left(LD\right)}\\ P_{13}=-Np\frac{sin\left(LD/2\right)}{LD/2}\frac{\left(\Delta \alpha^{*}\;+\;\kappa^{*}\alpha \right)sin\left(LD/2\right)\;-\;j\alpha^{*}Dcos\left(LD/2\right)}{Dcos\left(LD\right)\;+\;j\Delta sin\left(LD\right)}\\ P_{23}=-{\left(-1\right)}^{2N}Np\frac{sin\left(LD/2\right)}{LD/2}\frac{\left(\Delta \alpha \;+\;\kappa \alpha^{*}\right)sin\left(LD/2\right)\;-\;j\alpha Dcos\left(LD/2\right)}{Dcos\left(LD\right)\;+\;j\Delta sin\left(LD\right)}\\ P_{31}=-2P_{13}\\ P_{32}=-2P_{23}\\ P_{33}=-\frac{4}{D^{3}}\frac{\left[\left(\Delta^{2}\;+\;{\left|\kappa \right|}^{2}\right){\left|\alpha \right|}^{2}\;+\;2\Delta Re\left(\kappa^{*}\alpha^{2}\right)\right]\left[1\;-\;cos\left(LD\right)\right]}{Dcos\left(LD\right)\;+\;j\Delta sin\left(LD\right)}\;+\;\\ j\frac{4}{\Delta^{2}\;-\;{\left|\kappa \right|}^{2}}\frac{\left[\Delta {\left|\alpha \right|}^{2}\;+\;Re\left(\kappa^{*}\alpha^{2}\right)\right]sin\left(LD\right)}{Dcos\left(LD\right)\;+\;j\Delta sin\left(LD\right)}-j4L\frac{\Delta {\left|\alpha \right|}^{2}\;+\;Re\left(\kappa^{*}\alpha^{2}\right)}{\Delta^{2}-{\left|\kappa \right|}^{2}}\;+\;jL\omega C, \end{array}\;$$

where $D=\sqrt{{∆}^{2}\;-\;{\left|\kappa \right|}^{2}}$, and *L* denotes the length of the device.

Furthermore, since practical SAW devices are typically composed of multiple transducers and reflection gratings, it is necessary to sequentially cascade each *P*-matrix unit to calculate device response. Therefore, let *P^A^* and *P^B^* be the neighboring *P*-matrices cascaded from left to right; the cascading formula for the *P*-matrix is expressed as follows [10]:

(5) $$\begin{array}{l} P_{11}=P_{11}^{A}+P_{11}^{B}\frac{P_{21}^{A}P_{12}^{A}}{1\;-\;P_{11}^{B}P_{22}^{A}}\\ P_{12}=P_{21}=\frac{P_{12}^{A}P_{12}^{B}}{1\;-\;P_{11}^{B}P_{22}^{A}}\\ P_{22}=P_{22}^{B}+P_{22}^{A}\frac{P_{12}^{B}P_{21}^{B}}{1\;-\;P_{11}^{B}P_{22}^{A}}\\ P_{13}=P_{13}^{A}+P_{12}^{A}\frac{P_{13}^{B}\;+\;P_{11}^{B}P_{23}^{A}}{1\;-\;P_{11}^{B}P_{22}^{A}}\\ P_{23}=P_{23}^{B}+P_{21}^{B}\frac{P_{23}^{A}\;+\;P_{22}^{A}P_{13}^{B}}{1\;-\;P_{11}^{B}P_{22}^{A}}\\ P_{33}=P_{33}^{A}+P_{33}^{B}+P_{32}^{A}\frac{P_{13}^{B}\;+\;P_{11}^{B}P_{23}^{A}}{1\;-\;P_{11}^{B}P_{22}^{A}}+P_{31}^{B}\frac{P_{23}^{A}\;+\;P_{22}^{A}P_{13}^{B}}{1\;-\;P_{11}^{B}P_{22}^{A}} \end{array}$$

As for a single-port resonator without reflectors, *P*_33_ represents the admittance of the interdigital transducers (IDT). For this case, the boundary conditions are given by *a*_1_ = *a*_2_ = 0, and *P*_33_ represents the input admittance of the SAW resonator.

### 2.2. COM Parameter Extraction

**Figure 2:**
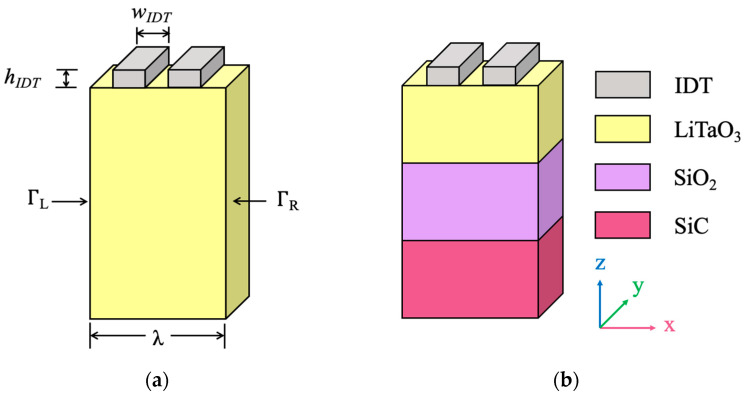
The schematic of the FEM model and structure used for simulation. (**a**) A SAW resonator on a 36°YX−LT substrate, and (**b**) a SAW resonator on a 36°YX−LT/SiO_2_/SiC substrate.

A theoretical method to extract precise COM parameters based on FEM software COMSOL Multiphysics 5.5 is presented. Figure 2 shows the schematic of a quasi-three-dimensional periodic FEM model and a structure used for simulation. As shown, one period with periodic metal electrodes alternately applying a positive or negative voltage is considered, and a perfectly matched layer (PML) is applied to the bottom to reduce the model’s size and suppress the unwanted boundary reflection. Periodic boundary conditions were defined on the left (Γ_L_) and right (Γ_R_) of the model. In addition, the meshing grid elements in each domain of the model are applied, and the maximum size of the grid cells in each material is specified as one-sixteenth of the wavelength to accurately resolve the stress waves in the solid domain. Furthermore, each IDT requires at least three solid cells in the thickness direction to ensure the accuracy of the calculation. The details of the quasi-3D periodic FEM model are reported in our previous work [24]. The piezoelectric module involving the physical field of solid mechanic and electrostatic coupling with the temperature field is performed so that the temperature-dependent COM parameters can be obtained. For a given temperature (T), the thermal effect can be computed by incorporating the linear thermal expansion coefficients and temperature coefficients of elasticity into the model.

**Figure 3:**
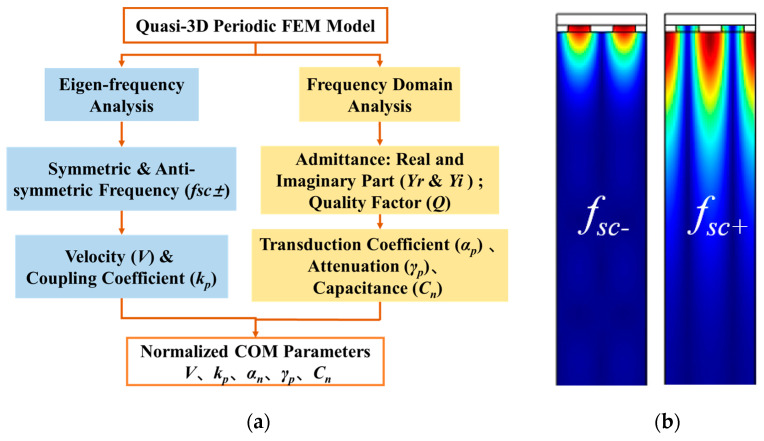
The FEM analysis with quasi-3D periodic structures: (**a**) the procedure of COM parameter extraction; (**b**) modal displacements at the stopband edges.

Figure 3 shows the procedure for the extraction of COM parameters. As is shown, an eigen frequency and frequency domain analysis of the multiphysics quasi-3D periodic FEM model for the SAW resonator is performed. As shown, the symmetric frequency (*f_sc−_*) and anti-symmetric frequency (*f_sc+_*) can be obtained by performing eigen frequency analysis at short-circuited grating, and then the center frequency ($f_{0}$), *v*, and *κ_p_* can be calculated using the following:

(6) $$f_{0}=\frac{f_{sc+}+f_{sc-}}{2},$$

(7) $$v={\lambda f}_{0},$$

(8) $$\kappa_{p}=2\pi \left(\frac{f_{sc-}}{f_{0}}-1\right).$$

Frequency domain analysis of the proposed model allows for the determination of the admittance, *Y*, and *Q* factor of the SAW resonator, which can be calculated via harmonic analysis under the condition where a sinusoidal signal with voltage, *V*/2, is applied to the electrode. Then, the harmonic admittance, *Y*, per IDT period is estimated using $Y=2\pi fjQ_{c}/V$, where *f* is the driving frequency and $Q_{c}$ is the total charge induced on the electrode [24]. Therefore, the remaining COM parameters, *α_p_*, *γ_p_*, and *C_p_*, can be obtained using the following formulas:

(9) $$\alpha_{p}=\sqrt{\frac{Y_{r}\gamma_{p}}{4}},$$

(10) $$\gamma_{p}=\pi \frac{∆f}{f_{0}}\;,$$

(11) $$C_{p}=\frac{2Y_{r}Q(\frac{f_{ar}}{f_{sc-}}-1)}{2\pi f_{ar}[4Q^{2}{\left(\frac{f_{ar}}{f_{sc-}}-1\right)}^{2}+1]}\;\;,$$

where $Y_{r}$ is the conductance at the resonant frequency, ∆f is the half-peak width of the conductance, $f_{ar}$ is the anti-resonant frequency, and the quality factor is given by $Q=\frac{f_{sc-}}{∆f}$. When the above five COM parameters are available, the device response and temperature behavior of the SAW device can be calculated.

### 2.3. Model Verification

For validation, first, the COM parameters extracted using the proposed method are verified for the normal SAW resonator based on bulk 128°YX−LiNbO_3_ substrate. Due to the almost negligible attenuation of Rayleigh waves excited on LiNbO_3_ substrates, the attenuation coefficient was not extracted. Table 1 presents a comparison between the extracted COM parameters and those of the previously reported results taken from Refs. [25,26] in our previous work [27]. As can be seen, these extracted COM parameters at a temperature (T) of 25 °C are compared well. Additionally, the COM parameters at a temperature (T) of 100 °C are also calculated via the proposed method. It is obvious that the temperature-dependent COM parameters vary with the temperature change. Compared to the traditional COM and pure FEM methods, the proposed method takes into account the temperature effects, allowing for a combination of faster and more accurate consideration of practical influences when designing SAW devices in the manufacturing process. This enhances the alignment between the expected and achieved outcomes.

**Figure 4:**
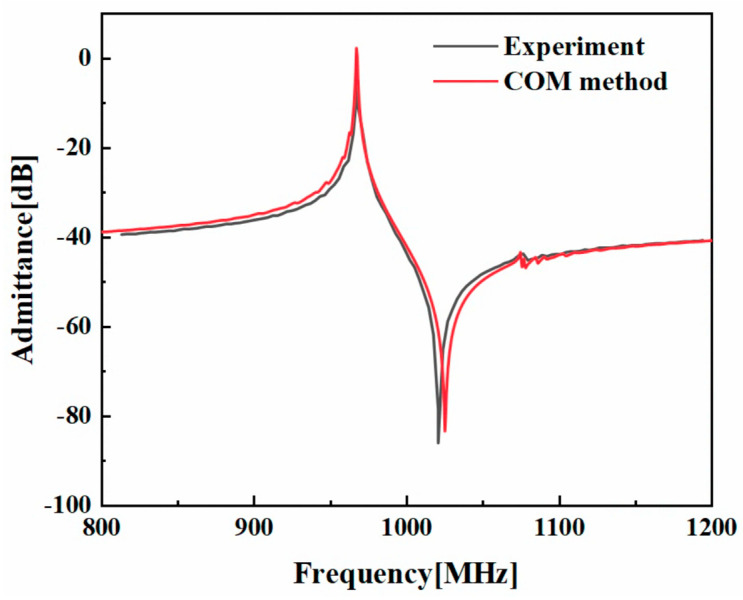
An admittance comparison between the proposed COM method and measured results [28] for the SAW resonator on a 36°YX−LT/SiO_2_/SiC multi-layered substrate.

Then, the extracted COM parameters are employed to simulate the admittance of the SAW device with the *P*-matrix model. Figure 4 presents the calculated admittance of the multi-layered SH-SAW resonator on a 36°YX−LT/SiO_2_/SiC layered substrate. For comparison, the measurement taken from Ref. [28] is also presented. As shown, the admittance curves basically fit well, and there exists a small difference at the anti-frequency, which is mainly caused by a bulk wave. Nevertheless, it is noted that the proposed COM method accurately reproduces fluctuations at low frequencies and a spurious response at high frequencies. 

## 3. Results and Discussion

### 3.1. Simulation of Resonators

Based on the advantages of fast and accurate simulations offered by the COM method, this work employs the COM method for the simulation and optimization of SAW resonators and DMS filters. 

**Figure 5:**
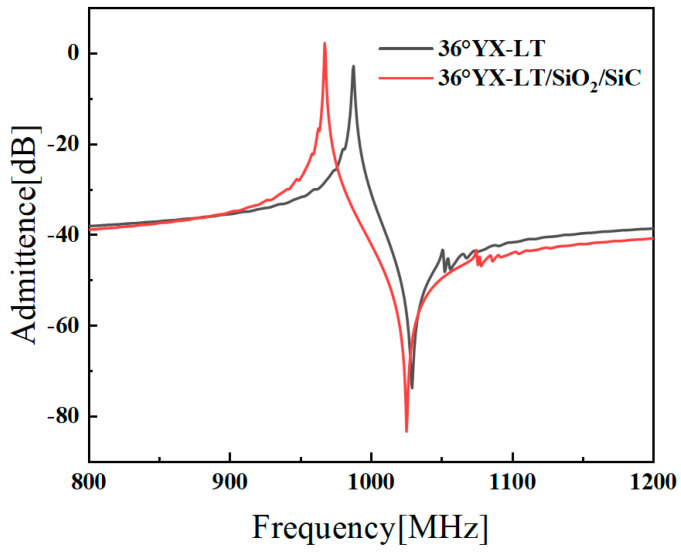
The admittance comparison between the SH-SAW resonator with bulk 36°YX−LT substrate and the SH-SAW resonator with the 36°YX−LT/SiO_2_/SiC multi-layered substrate.

Figure 5 shows the calculated admittance of resonators for both bulk 36°YX−LT substrate and the 36°YX−LT/SiO_2_/SiC multi-layered structure. The structural parameters of the resonator are shown in Table 2. It can be observed that the multi-layered structure exhibits significantly higher *K*^2^ and a larger oscillation amplitude. This can be attributed to the SiC substrate in the multi-layered structure, which has a higher velocity and thus offers an SAW energy confinement effect. This characteristic allows for the efficient conversion of electrical energy into mechanical vibration, enabling the high performance of SAW devices. Compared to the bulk LT substrate, the LT/SiO_2_/SiC multi-layered substrate has advantages in terms of a higher *Q* and improved temperature stability. From this point on, the 36°YX−LT/SiO_2_/SiC multi-layered structure was further optimized in terms of different Euler angles and geometric structural parameters.

The advantages of the multi-layered structure over those of a bulk LT structure, in terms of SAW resonator fabrication, typically result in a higher *Q* and improved temperature stability. Therefore, in this study, the multi-layered structure is optimized and employed for the simulation of SAW devices.

**Figure 6:**
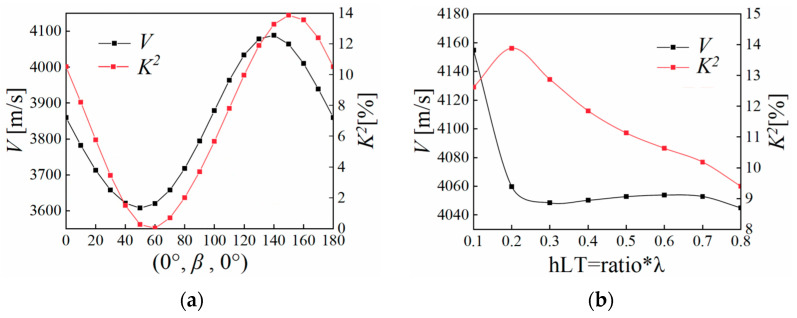

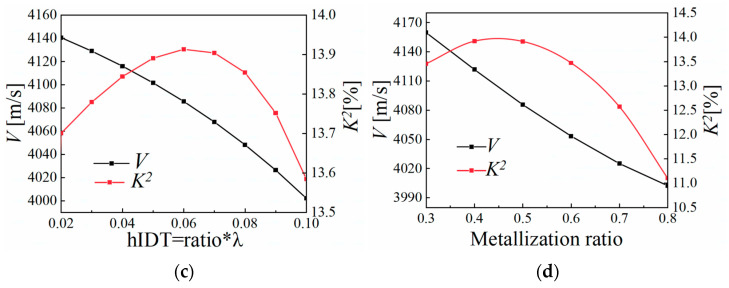
The calculated velocities and electromechanical coupling coefficients of the multi-layered structure versus the (**a**) *β* angles of the LT layer. The LT layer thickness is 800 nm. The metallization ratio is 0.5. The electrode thickness is 297 nm. (**b**) Piezoelectric layer thicknesses; *β* = 151°. (**c**) Electrode thicknesses; *β* = 151°. (**d**) Metallization ratios.

Figure 6a presents the calculated velocities and electromechanical coupling coefficients of an SH wave mode on the LT/SiO_2_/SiC structure versus different *β* rotation angles of the LT layer. The maximum *K*^2^ value of the device, which was approximately 13.88%, was achieved by setting the Euler angle of the LT layer in the COMSOL as (0°, 151°, 0°) when the LT layer thickness at 800 nm. In this configuration, the phase velocity reached around 4064 m/s.

Additionally, Figure 6b illustrates the calculated velocities and electromechanical coupling coefficients versus different piezoelectric layer thicknesses on a 29°YX−LT/SiO_2_/SiC structure. A *K*^2^ value of 13.89% is obtained when the piezoelectric layer thickness is 0.2λ, with a corresponding phase velocity of 4060 m/s. This reduction in velocity is deemed acceptable compared to the case for a piezoelectric layer thickness of 0.1λ. In the case of a 0.2λ thick LT layer, a *K*^2^ value of 13.91% is obtained when the electrode thickness is 0.06λ, as shown in Figure 6c. It is worth noting that as the electrode thickness increases, the velocity of the SH wave mode gradually decreases due to the influence of mass loading. However, the velocity of 4085 m/s at the electrode thickness of 0.06λ is an acceptable value. Under the condition of a 0.2λ thick LT layer and 0.06λ thick Al electrodes, an optimized structure with a maximum *K*^2^ of 13.92% and suitable phase velocity of 4122 m/s obtained simultaneously results when the metallization ratio is 0.4.

**Figure 7:**
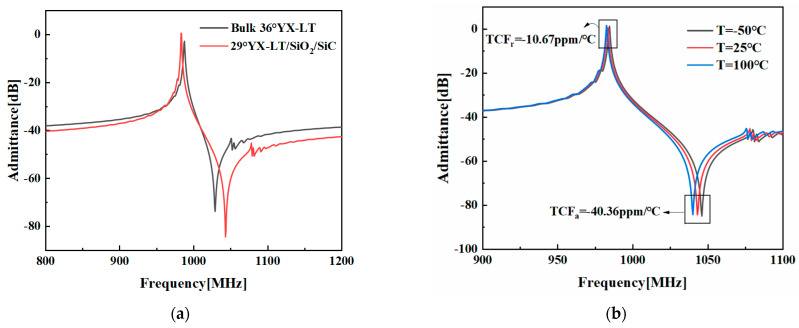
(**a**) The admittance comparison between the SH-SAW resonator on the 36°YX−LT substrate and the SH-SAW resonator on the optimized 29°YX−LT/SiO_2_/SiC structure. (**b**) Admittances of the optimized 29°YX−LT/SiO_2_/SiC structure at different temperatures.

Figure 7a presents the simulation results of the optimized resonator compared to those of a resonator on a 36°YX−LT substrate. The optimized resonator on the 29°YX−LT/SiO_2_/SiC structure exhibits significantly larger *K*^2^ values and higher amplitudes compared to the resonator on a 36°YX−LT substrate. Figure 7b illustrates the temperature characteristics of the optimized resonator on the 29°YX−LT/SiO_2_/SiC structure. It is evident that as the temperature increases, the overall admittance gradually shifts to the left. The temperature coefficient of the resonance frequency (TCF_r_) is −10.67 ppm/°C, while the temperature coefficient of the anti-resonance frequency (TCF_a_) is −40.36 ppm/°C [29,30,31]. Table 3 presents a comparison for the extracted COM parameters at different temperatures. As can be seen, these extracted temperature-dependent COM parameters vary with the temperature change. The performance comparison between the resonators on a 36°YX−LT substrate and a 29°YX−LT/SiO_2_/SiC substrate is shown in Table 4. The multi-layered structure demonstrates a significant improvement in temperature stability, which effectively mitigates the impact of temperature variations on SAW resonators and meets the requirements for precise and stable performance in practical applications.

### 3.2. Simulation of DMS Filters

The advantages of the optimized 29°YX−LT/SiO_2_/SiC structure over a 36°YX−LT structure typically imply a larger bandwidth and lower insertion loss for filters. Therefore, this work extends the optimization results of multi-layered SAW resonators and applies the optimized structure for a DMS filter simulation. 

**Figure 8:**
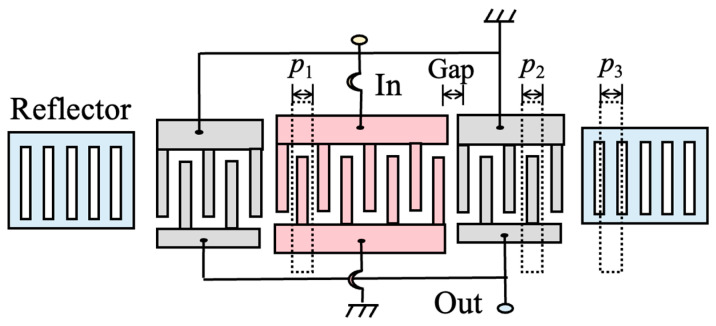
Configuration of a test DMS structure using the first mode and third mode.

In order to obtain a wider bandwidth, the test DMS structure of three IDTs in which the lateral IDTs are symmetrically arranged with respect to the center IDT is used [32]. Figure 8 presents the electrode configuration. The corresponding parameters of DMS filters are shown in Table 5.

**Figure 9:**
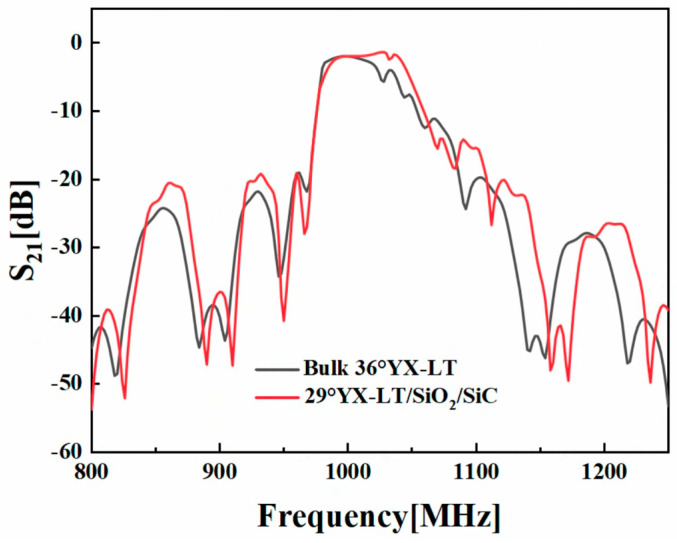
The transmission spectrum comparison between the DMS filters on the bulk 36°YX−LT substrate and the optimized 29°YX−LT/SiO_2_/SiC structure.

Figure 9 illustrates a comparison of the transmission spectrums between the filters on the 36°YX−LT and 29°YX−LT/SiO_2_/SiC substrates. The DMS filter on the 29°YX−LT/SiO_2_/SiC substrate exhibits a significantly wider bandwidth compared to that of the common filter. This expanded bandwidth enables the filter to transmit a broader range of frequencies, meeting the requirements for processing various frequency signals. Additionally, the 29°YX−LT/SiO_2_/SiC structure demonstrates a flatter passband, ensuring uniform signal transmission and avoiding an uneven frequency response and distortion. This is crucial for applications that demand good signal quality and accuracy. Therefore, the DMS filter on the 29°YX−LT/SiO_2_/SiC substrate provides more flexible and high-performance signal processing capabilities.

**Figure 10:**
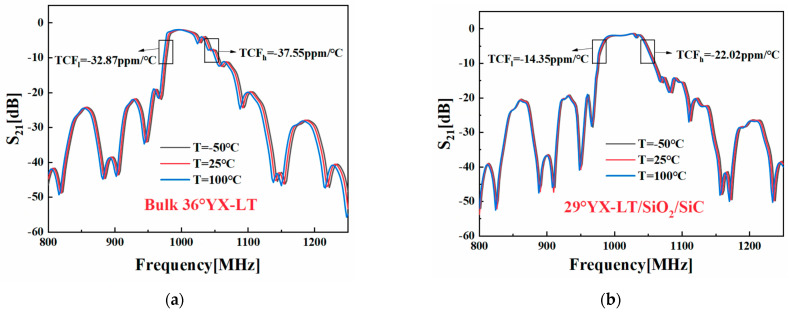
Simulated transmission spectrums of DMS filters with (**a**) the bulk 36°YX−LT substrate and (**b**) the optimized 29°YX−LT/SiO_2_/SiC structure at different temperatures.

Figure 10 illustrates the computed temperature characteristics of the two DMS filters, indicating that the optimized 29°YX−LT/SiO_2_/SiC structure exhibits superior temperature stability compared to that of the 36°YX−LT structure. Particularly, TCF suppression is evident at lower frequencies in the 29°YX−LT/SiO_2_/SiC structure. TCF values at the lower edge (TCF_l_) are reduced to −14.35 ppm/°C, while TCF values at the higher edge (TCF_h_) are reduced to −22.02 ppm/°C compared to those of the DMS filter on a 36°YX−LT structure (TCF_l_ = −32.87 ppm/°C, TCF_h_ = −37.55 ppm/°C). The 29°YX−LT/SiO_2_/SiC structure effectively suppresses the TCF to a satisfactory level, emphasizing its contribution to improved temperature stability. Table 6 presents the specific performance of the two DMS filters. In addition to the improvement in TCF, in-band insertion loss is optimized from −3 dB to −2 dB, effectively enhancing the application potential of the filter at high temperatures and enabling the better performance of signal transmission. It can be inferred that by employing the improved method, it is possible in the early stages of designing SAW devices to simulate the propagation characteristics and temperature properties of DMS filters rapidly and accurately in the manufacturing process. This facilitates the identification of filters that meet the expected requirements, enabling mass production and reducing material wastage caused by inaccurate or incomplete simulations.

## 4. Conclusions

In this paper, an advanced method combining the COM theory with a quasi-3D periodic FEM model coupled with a thermal field is proposed. Accurate simulations and analyses of the electroacoustic and temperature behavior of SAW resonators and filters are performed. This is of great assistance in the manufacturing process of SAW devices, effectively preventing production wastage caused by inaccurate simulations or the neglect of temperature stability. On this basis, a comparison is made between resonators on a 36°YX−LT structure and those on a 29°YX−LT/SiO_2_/SiC multi-layered structure. This comparison demonstrates the advantages of the multi-layered structure with a larger *K*^2^ of 13.92% and a lower TCF of −10.67 ppm/°C. Frequency response calculations are performed for both the SAW resonators and filters based on this optimized structure, revealing significant improvements in performance compared to that of a normal structure. In particular, the temperature stability and the in-band insertion loss of the optimized DMS filter are improved, highlighting the tremendous potential of SAW filters in high-temperature applications.

## Figures and Tables

**Table 1:** COM parameters for 128°YX−LiNbO_3_ with *h_IDT_* = 0 [27].

Parameters	Ref. [25]	Ref. [26]	This Work	
T [℃]	25	25	25	100
v [m/s]	3899.98	3901	3899	3876
$\kappa_{p}\;[\%]$	−3.95	−3.95	−3.89	−3.93
$\alpha_{n}\;[{10^{-5}\;\Omega }^{-\frac{1}{2}}]$	68.618	69.65	69.81	70.15
$C_{n}\;[10^{-5}\;pF/\mu m]$	49.263	48.36	49.3	48.3

**Table 2:** The geometric structure parameters of the SAW resonator.

Parameters	Value
Wavelength (*λ*)	4 (μm)
Aperture	15 ∗ λ
IDT thickness	0.06 ∗ λ
Metallization ratio	0.4
Number of IDT finger pairs	75
Number of reflector strips	40

**Table 3:** COM parameters for the 29°YX−LiTaO_3_ structure.

Parameters	T = −50 °C	T = 25 °C	T = 100 °C
v [m/s]	4128	4122	4116
$\kappa_{p}\;[\%]$	−28.94	−28.66	−28.19
$\alpha_{n}\;[{10^{-5}\;\Omega }^{-\frac{1}{2}}]$	86.93	86.62	85.83
$C_{n\;}[10^{-5}\;pF/\mu m]$	29.30	29.91	30.73
$\gamma_{p}\;[/]$	0.00231	0.00232	0.00232

**Table 4:** Performance comparison between the SAW resonators on the 36°YX−LT substrate and 29°YX−LT/SiO_2_/SiC substrate.

Structure Configuration	*K*^2^ (%)	*V* (m/s)	TCF_r_ (ppm/°C)	TCF_a_ (ppm/°C)	*Q_r_*
Bulk 36°YX−LT	10.16	4075	−30.21	−48.01	773
29°YX−LT/SiO_2_/SiC	13.92	4122	−10.67	−40.36	1265

**Table 5:** The geometric structure parameters of the DMS filter.

Parameters	Value
*p*_1_	2 [μm]
*p*_2_	1.9 [μm]
*p*_3_	2.3 [μm]
Gap	1 [μm]
IDT finger pairs of the input part	15.5
IDT finger pairs of the output part	9
Number of reflector strips	60

**Table 6:** Performance comparison between the two DMS filters based on a 36°YX−LT substrate and a 29°YX−LT/SiO_2_/SiC substrate.

Structure Configuration	Insert Loss (dB)	TCF_l_ (ppm°C^−1^)	TCF_h_ (ppm°C^−1^)
Bulk 36°YX−LT	−3	−32.87	−37.55
29°YX−LT/SiO_2_/SiC	−2	−14.35	−22.02

## Data Availability

Data are contained within the article.
